# Generic and disease-specific quality of life among youth and young men with Hemophilia in Canada

**DOI:** 10.1186/s12878-016-0052-x

**Published:** 2016-05-05

**Authors:** J. St-Louis, D. J. Urajnik, F. Ménard, S. Cloutier, R. J. Klaassen, B. Ritchie, G. E. Rivard, M. Warner, V. Blanchette, N. L. Young

**Affiliations:** CHU Sainte-Justine, Montréal, Canada; Hôpital Maisonneuve-Rosemont, Montreal, Canada; Laurentian University, Sudbury, Canada; Hôpital de l’Enfant-Jésus, Quebec city, Canada; University of Alberta, Edmonton, Canada; Children’s Hospital of Eastern Ontario, Ottawa, Canada; McGill University Health Centre, Montréal, Canada; University of Toronto, Toronto, Canada; Hospital for Sick Children, Toronto, Canada

**Keywords:** Hemophilia, Health-related quality of life, Canadian population, Prophylaxis

## Abstract

**Background:**

This study was undertaken to explore the longitudinal patterns of health-related quality of life (HRQoL) among youth and young adults with Hemophilia A (HA) over a 3-year period. This report presents the baseline characteristics of the study cohort.

**Methods:**

Males, 14 to 29 years of age, with predominantly severe HA were recruited from six treatment centres in Canada. Subjects completed a comprehensive survey. HRQoL was measured using: the CHO-KLAT_2.0_ (youth), Haemo-QoL-A (young adults) and the SF-36v2 (all).

**Results:**

13 youth (mean age = 15.7, range = 12.9-17.9 years) and 33 young adults (mean age = 23.6; range = 18.4 -28.7 years) with moderate (7 %) and severe (93 %) HA were enrolled. All were on a prophylactic regimen with antihemophilic factor (Helixate FS®) during the study. The youth had minimal joint damage (mean HJHS = 5.2) compared to young adults (mean HJHS = 13.3). The mean HRQoL scores for youth were: 79.2 (SD = 11.9) for the CHO-KLAT, and 53.0 (5.5) and 52.3 (6.8) for the SF-36 Physical Component Summary (PCS) and Mental Component Summary (MCS) scores respectively. The mean HRQoL scores for young adults were: 85.8 (9.5) for the Haemo-Qol-A, and 50.8 (6.4) and 50.9 (8.8) for PCS and MCS respectively. PCS and MCS scores were comparable to published Canadian norms, however significant differences were found for the domains of Physical Functioning and Bodily Pain. The disease-specific HRQoL scores were weakly correlated with the PCS for youth (CHO-KLAT vs. PCS *r* = 0.28, *p* = 0.35); and moderately correlated for the MCS (*r* = 0.39, *p* = 0.19). Haemo-QoL-A scores for young adults were strongly correlated with the PCS (*r* = 0.53, *p* = 0.001); and weakly correlated with the MCS (*r* = 0.26, *p* = 0.13). Joint status as assessed by HJHS was correlated with PCS scores. A history of lifelong prophylaxis resulted in better PCS but worse MCS scores.

**Conclusion:**

Despite having hemophilia, the youth in this cohort have minimal joint disease and good HRQoL. The young adults demonstrated more joint disease and slightly worse HRQoL in the domains of physical functioning and pain. The data presented here provide new information to inform the selection of Health Related Quality of Life (HRQoL) instruments for use in future clinical trials involving persons with hemophilia.

**Trial registration:**

ClinicalTrials.gov : NCT01034904. Study funded by CSL Behring Canada.

**Electronic supplementary material:**

The online version of this article (doi:10.1186/s12878-016-0052-x) contains supplementary material, which is available to authorized users.

## Background

Hemophilia A is a hereditary disorder resulting in deficient levels of plasma coagulation factor VIII (FVIII) and lifelong bleeding manifestations, particularly repeated hemarthroses leading to permanent joint damage. Major progress has been demonstrated in preventing bleeding and disability when intensive factor replacement therapy is administered prophylactically from an early age [1]. The objective assessment of the outcome of different treatment strategies in hemophilia is multidimensional and includes the measurement of bleeding rates, joint and musculoskeletal status, pain, physical functioning and social functioning based on holistic models of health as recommended by the WHO [2]. Health-related quality of life (HRQoL) tools are self-administered questionnaires developed in order to measure the perceived impact of a medical condition and its treatment on a person’s physical, emotional and social well-being.

In hemophilia both generic and disease-specific HRQoL questionnaires have been used in recent years although seldom simultaneously in the same subjects. Recent clinical trials have incorporated the measurement of HRQoL scores before and after an intervention such as the introduction of a prophylactic factor replacement regimen. However, there is very little published data on long term longitudinal trends of HRQoL in hemophilia. Significant variations in HRQoL may not be determined only by change in physical status but may be influenced by common social events such as educational, vocational or relational changes associated with transition from adolescence to adulthood.

We initiated a study in which HRQoL was assessed and described prospectively every 6 months over a 3-year period in a cohort of youth and young adults with severe or moderate hemophilia A receiving routine care. This study will enable the assessment of the relationship between changes in HRQoL scores and changes in physical health as evaluated by clinical assessments (bleeding frequency, product utilization and joint scores). It will also enable us to examine the sensitivity of the main HRQoL measures to significant life events in patients with hemophilia. We present in this report the baseline information on HRQoL for the cohort of Canadian youth and young adults with HA that were followed prospectively over a 3-year period.

## Methods

### Recruitment/Sample

To be included in the study subjects were required to be males 14 to 29 years of age with moderate (FVIII level 0.02 – 0.05 U/ml) or severe HA (FVIII level < 0.02 U/ml) treated with the recombinant antihemophilic factor Helixate FS® either on a prophylactic or “on-demand” regimen. Subjects were identified from the clinical records at six hemophilia treatment centres in Canada, three in the province of Quebec (Montreal and Quebec City) and three outside (Edmonton, Toronto, Ottawa). Potential subjects were excluded if they had a current inhibitor to FVIII defined as an inhibitor level of equal to or greater than 0.6 Bethesda Units/mL, human immunodeficiency virus (HIV) infection or symptomatic hepatitis C virus (HCV) infection. Information on ethics approval and consent is provided in the Declarations section below. Study funding was provided by CSL Behring Canada. The study was registered in the ClinicalTrials.gov database on December 17, 2009 under the trial number: NCT01034904.

### Measures/Manoeuvre

#### Chart review and demographic survey

The following baseline data was obtained by chart review: severity of haemophilia, history of target joints in the preceding year (defined as a joint with 3 or more bleeds in 3-month period), history of prior major bleeding events requiring hospital admission (e.g. an intracranial hemorrhage), previous surgery, current treatment program, and any concomitant medical condition. Definitions of a target joint and prophylaxis as stated in the protocol were based on published Canadian consensus definitions [3]. Subjects also completed a comprehensive demographic survey relating to educational and professional experience.

#### Health- related quality of life assessment

All subjects completed a generic and a disease-specific HRQoL questionnaire at baseline.

The same generic questionnaire was used for all subjects. The Medical Outcomes Study 36-item Short Form health survey (SF-36) is an instrument that has been validated for a variety of diseases and has been widely used in hemophilia. It has been normed for populations in several countries and therefore allows comparisons with both normal and diseased populations [4]. The 36 questions are grouped to assess 8 domains of HRQoL with scores ranging from 0 (worse) to 100 (best): Physical Functioning (PF), Role Physical (RP), Bodily Pain (BP), General Health (GH), Vitality (VT), Social Functioning (SF), Role Emotion (RE) and Mental Health (MH). Furthermore, the Physical Component Summary (PCS) and Mental Component Summary (MSC) scores are derived from the 8 domains. These are reported using norm-referenced scoring with a mean of 50 and standard deviation of 10 points [5].

Subjects completed a different disease-specific HRQoL questionnaire depending on their age. Youth aged 14 to 17 years completed the Canadian Haemophilia Outcomes – Kids Life Assessment Tool (CHO-KLAT) version 2.0 [6–9], which was developed and validated for children and adolescents with hemophilia. It consists of 35 questions and is scored from 0 to 100.

In subjects 18 years and above the Haemo-QoL-A, developed by Rentz et al, was used [10]. It comprises 41 items grouped into 6 subscales, each scored from 0 (worst) to 100 (best): Physical Functioning, Role Functioning, Worry, Consequences of Bleeding, Emotional Impact and Treatment Concerns. This disease-specific instrument has also been demonstrated to be reliable and valid for assessing HRQoL in adult patients in hemophilia clinical trials [10].

#### Joint status assessment

A standardized physical examination of the joints most commonly affected by hemophilia (the elbows, knees and ankles) was performed by a trained physiotherapist using the Hemophilia Joint Health Score (HJHS) version 2.0 [11–13]. This comprises an assessment of each of these 6 joints with regards to swelling, muscle atrophy, crepitus, range of motion, joint pain, strength, and global gait. The score for each joint is summed to obtain a total score ranging from 0 to 124, where no joint damage is indicated by a score of 0. A total score above 10 has been considered indicative of significant joint disease in a published study of young adults [14].

### Statistical analysis

Descriptive statistics were generated for the demographic, clinical, and HRQoL measures. Frequencies and/or percentages (%) are reported for categorical data. Means and standard deviations (SD) are presented for continuous measures that are normally distributed. Medians and ranges are presented for skewed data (skew or kurtosis ratio > ±3.0). Benchmarks from the literature were included to enable comparisons.

The scores from generic and disease specific HRQoL measures were compared using Pearson correlations. Pearson correlations were also used to examine the relationship between HRQoL and joint status as measured by the HJHS. We interpreted the strength of each correlation as recommended by Cohen, with a correlation of 0.1 indicating a weak relationship, 0.3 to 0.5 indicating a moderate relationship, and above 0.5 indicating a large or strong relationship [15].

The relationships between disease characteristics, treatment program, joint status and HRQoL were explored using independent sample t-tests and analysis of variance (ANOVA). The incremental impact of age on the PCS and MCS SF-36 summary scores was tested via linear multiple regression controlling for joint status as measured by the HJHS. The minimally important difference (MID) threshold for the SF-36 was used as the criterion for clinical significance on this generic measure. MID’s are reported for the PCS (2–3), MCS (3), PF (2–3), RP (2), BP (2–3), GH (2–3), VT (2–3), SF (3), RE (4), and MH (3) [5]. Score differences above the MID are considered meaningful. Minimal thresholds for changes in scores that are clinically relevant have not been defined for CHO-KLAT and Haemo-QoL-A. Stata® version 13.0 was used to perform all analyses.

## Results

Forty-eight subjects were enrolled into this longitudinal study however two subjects were excluded from the analysis due to incomplete data and withdrawal of consent. The sample reported here included: 13 youth (mean age = 15.7, range = 12.9–17.9 years) and 33 young adults (mean age = 23.6; range = 18.4–28.7 years). One 12.9 year-old subject was included in the analysis. His recruitment was a protocol deviation allowed by the investigators due to the small number of youth in this study. The group included 43 patients with severe disease (93 %); as well as 2 youth and one adult with moderate disease.

At recruitment, 15 % of the youth and 27 % of the young adults were considered to have had an active target joint in the prior year. Of these, 2 young adults had more than one target joint. HJHS scores ranged from 0 to 17 in the youth (mean = 5.2, standard deviation, SD = 5.61) and 0 to 34 in the young adults (mean = 13.3, SD = 8.93), where no joint damage is indicated by a score of 0. Significant joint disease (HJHS scores >10) was more common among young adults (64 %) as compared to youth (23 %).

All subjects were on some form of prophylaxis at the time of recruitment, which was defined as the regular infusion of FVIII at least once weekly with the aim of preventing clinically significant bleeding [3]. Although eligible, no subjects on an “on demand” regimen were recruited because of the paucity of patients on such a regimen in the specified age group at the study sites. Ten subjects (8 youth and 2 adults) were considered by the investigators to have been on lifelong primary prophylaxis from early childhood until the time they were recruited to the study. For the purpose of this study, primary prophylaxis was defined as prophylaxis that was started in a patient with no established joint disease, usually in the first or second year of life, before a third bleed but usually after a first bleed [3]. In our sample, 8 subjects (17.4 %) were currently on prophylaxis one to two times per week, 21 (45.6 %) were on 3x/week or alternate day prophylaxis and 17 (36.9 %) were on daily prophylaxis. These details are summarized in Table 1.

**Table 1:** Baseline sample characteristics

	Study cohort youth	Study cohort young adults
Sample Size	13	33
Ages (years)	Mean = 15.6	Mean = 23.6
	SD = 1.4	SD = 2.9
	Range: 12.9 to 17.9	Range: 18.4 to 28.7
Proportion with Severe Haemophilia	85 %	97 %
Proportion with a Target Joint	15 %	27 %
Proportion on Prophylaxis	100 %	100 %
Prophylaxis Frequency	Once or Twice a week = 15 %	Once or Twice a week = 18 %
	Alternate Days = 54 %	Alternate Days = 42 %
	Daily = 31 %	Daily = 39 %
HJHS	Mean = 5.2	Mean = 13.3
	SD = 5.6	SD = 8.9
	Range: 0 to 17	Range: 0 to 34

The subjects were predominantly (83 %) from three centres in Quebec, a province where Helixate FS® was preferentially prescribed for public tender contractual reasons. Treatment protocols in this region do not differ in any meaningful way from protocols elsewhere in Canada.

Subjects had a mean body mass index (BMI) of 25.1 (SD = 4.80) with a range of 18.4 to 37.9, which is similar to the Canadian norm of 26.1 [16]. Based on 45 subjects with complete BMI data, none of the subjects were underweight, 56 % of the subjects were of normal weight, 24 % of them were overweight (BMI of 25 to 34.9) and 20 % of study subjects were obese (BMI ≥ 35).

The group was diverse in terms of occupation. Of 46 subjects, 9 (20 %) were in occupations that were physically demanding, 9 (20 %) were in jobs of relatively low physical demand, and 27 (60 %) were students. Among the students, 14 were in secondary school, 9 were attending college or a vocational school and 4 were attending university.

There were no major comorbidities in the study cohort. Seven patients had asymptomatic HCV. All other concomitant medical conditions were of mild severity (2 patients had asthma, and one each with hypertension, hypothyroidism and angioedema). One patient had a major depression more than 5 years prior, and 7 were considered to have suffered from attention deficit/hyperactivity disorder during childhood.

### HRQoL scores

Generic HRQoL scores (as measured by the SF36) were available for the whole cohort of 46 subjects, including the Component Summary scores (PCS and MCS), and scores for the 8 domains. Disease specific HRQoL scores were also available for the 13 youth (as measured by the CHO-KLAT) and 33 young adults (as measured by the Haemo-QoL-A).

In the combined cohort of 46 youth and young adults all core variables met the distributional assumptions necessary for parametric analyses. The PCS scores ranged from 35 to 63 with a mean of 51.4 (SD = 6.20), and MCS scores ranged from 32 to 64 with a mean of 51.3 (SD = 8.23). Details for the SF-36 results for youth and young adults are provided in Table 2. This table shows the distributions of scores for each group. Scores were slightly better in youth than in young adults on all scales, as would be expected based on the prevalence of target joints and arthropathy, with the exception of Social Functioning. In order to compare to published Canadian norms [17], we computed SF-36 scores for the sub-set of 27 subjects who were between the ages of 16 to 24.9 years at recruitment. These are presented on the right side of Table 2; mean differences between this group and the Canadian norms are also shown. When we examined the results in comparison to the Canadian norms, Physical Functioning and Bodily Pain scores were significantly worse in our hemophilia sample.

**Table 2:** SF36 score distributions and comparisons to Canadian Norms

Means (standard deviations)	Study Cohort Youth	Study cohort young adults	Study cohort comparative sub-set (selected to match the age range for published Canadian Norms [17])	Canadian Norms for Males [17]	Mean Difference
Ages (years)	12.9 to 17.9	18.4 to 28.7	16 to 24.9	16 to 24.9	
Sample Size	13	33	27	474	
PCS (norm-referenced scoring)	53.00	50.79	51.74	53.9	2.16
	(5.5)	(6.4)	(6.5)	(6.9)	
MCS (norm-referenced scoring)	52.3	50.9	51.1	49.3	-1.79
	(6.8)	(8.8)	(8.6)	(9.7)	
Physical Functioning	90.0	87.1	87.9	93.6	5.64*
	(10.4)	(13.2)	(13.7)	(13.3)	
Role Physical	86.1	84.1	84.5	89.9	5.41
	(17.2)	(13.9)	(14.9)	(27.1)	
Bodily Pain	75.9	66.2	69.2	79.1	9.9*
	(11.1)	(19.4)	(20.3)	(19.4)	
General Health	82.6	75.4	79.6	78.7	-0.93
	(13.6)	(15.5)	(13.9)	(14.7)	
Vitality	71.2	64.9	65.7	64.0	-1.74
	(10.4)	(16.4)	(16.9)	(16.5)	
Social Functioning	83.7	86.4	86.6	86.5	-0.07
	(16.4)	(12.6)	(14.3)	(18.7)	
Role Emotional	89.7	85.1	86.7	82.7	-4.03
	(14.5)	(18.9)	(17.5)	(32.8)	
Mental Health	80.4	77.1	77.2	74.3	-2.92
	(9.9)	(15.5)	(15.2)	(16.6)	

MID’s definitions: PCS =2-3; MCS = 3; PF = 2-3; RP = 2; BP = 2-3; GH = 2-3; VT = 2-3; SF = 3; RE = 4; and MH = 3 [5]

*p < 0.05

The CHO-KLAT scores for the youth ranged from 57.9 to 97.9 with a mean of 79.2 (SD = 11.86). The Haemo-QoL-A scores for the young adults ranged from 65.9 to 98.1 with a mean of 85.8 (SD = 9.54). Additional details of the Haemo-QoL-A scores in our adult cohort are presented in Table 3. This table also includes median scores from Manco-Johnson et al. [18], who reported results for a sample with a similar age range (all of whom were on prophylaxis) from the United States, and mean scores from Rentz et al. [10] based on an international sample of older patients with a range of disease severity and comorbidity. The overall Haemo-QoL-A scores in our cohort are similar to the US cohort with the exception of higher (better) scores for Treatment Concerns in the Canadians, and all of our scale scores were significantly higher than those reported by Rentz (*p* < 0.0001).

**Table 3:** Haemo-QoL-A score distributions and comparisons published results

	Study cohort young adults	United States Cohort Manco-Johnson Prophylaxis Group [18]	International Cohort Rentz [10]
Age (years)	Mean age23.6 (SD = 2.87) [range 18.4–28.7]	Median age 19.5 [range 14–29]	mean age 38.9 (SD = 14.7)
Proportion with Severe Haemophilia	93 %	100 %	52 %
Sample Size	*n* = 33	*n* = 21	*n* = 221
Total Score	85.8	85.6	73.1
	(9.53)	(10.7)	(16.96)
Physical Functioning	82.1	88.4	66.8
	(12.4)	(11.9)	(23.9)
Role Functioning	86.4	86.0	79.4
	(10.4)	(10.3)	(17.3)
Worry	82.4	85.5	73.6
	(17.1)	(15.3)	(24.2)
Consequences of Bleeding	87.5	86.7	72.2
	(10.9)	(9.4)	(21.9)
Emotional Impact	85.1	89.8	76.9
	(12.8)	(11.8)	(18.1)
Treatment Concerns	91.1	77.5	60.1
	(13.4)	(20.9)	(30.6)

#### Relationships between HRQoL measures

The relationships between the generic and disease-specific HRQoL scores were modest. The CHOK-LAT scores for the 13 youth had a weak correlation with the PCS (*r* = 0.28, *p* = 0.35), and moderate correlation with the MCS (*r* = 0.39, *p* = 0.19). The Haemo-QoL-A scores for the 33 young adults had a strong correlation with the PCS (*r* = 0.53, *p* = 0.001) and a weak correlation with the MCS (*r* = 0.26, *p* = 0.13). The lack of statistical significance for some of these observed relationships may be due in part to the small sample.

### Relationships between target joints and HRQoL

The interpretation of HRQoL scores may be aided by an understanding of how scores vary in relation to key clinical characteristics. Therefore, we explored the relationship between HRQoL scores and the presence of a target joint.

There was a clinically meaningful difference in PCS scores in the 11 subjects with one or more target joints (mean = 49.1; SD = 6.32) compared to those without a target joint (mean = 52.1; SD = 6.08). The MCS scores were minimally lower in the 11 subjects with a target joint (mean = 50.3; SD = 2.37) compared to those without a target joint (mean = 51.6; SD = 1.43). A difference of 3 points is considered clinically meaningful for the component scores [5] Neither of these differences were statistically significant (*p* = 0.18 and *p* = 0.66 respectively).

The Haemo-QoL-A scores did not reveal clinically meaningful or statistically significant differences between those with or without active target joints. We also explored the Physical Functioning sub-scale of the Haemo-QoL-A and found no difference. We were unable to assess the difference in CHO-KLAT scores associated with target joints, as there were only 2 youth with target joints.

### Relationships between joint status and HRQoL

Next, we explored the relationship between HRQoL scores relative to joint damage as measured by the HJHS. PCS scores were strongly correlated with HJHS scores (*r* = -0.62, *p* < 0.0001) in the combined sample (note: the correlations were similar for both the youth and adult groups). MCS scores had a weak correlation with HJHS scores (*r* = 0.12, *p* = 0.42). HJHS total scores were weakly correlated with both the CHO-KLAT scores in youth (*r* = -0.20, *p* = 0.52) and the Haemo-QoL-A scores in adults (*r* = -0.19, *p* = 0.28).

### Relationships between treatment and HRQoL

Finally, we explored the HRQoL scores in groups with different treatments. We began by looking specifically at the 10 subjects (including 8 youth) who had been on primary prophylaxis from early childhood compared to the 36 subjects who had not. The PCS scores for the 8 youth on primary prophylaxis (mean = 53.5; SD = 5.54) were only slightly higher than those for the 5 remaining youth (mean = 52.3 SD = 5.91). However, the MCS scores were worse for the youth on primary prophylaxis (mean = 49.9; SD = 7.70) when compared to the others (mean = 56.2; SD = 1.85). A similar trend was found for the two adults on primary prophylaxis (mean PCS = 53.8 SD = 9.79 and mean MCS = 36.6 SD = 4.29) compared to the 31 others (mean PCS = 50.6 SD = 6.35 and mean MCS = 51.8 SD = 8.23).

**Fig. 1:**
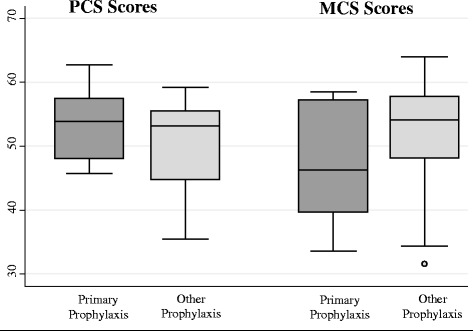
SF36 Component Scores by Prophylaxis Type

When we combined youth and adult groups the mean PCS for the primary prophylaxis group was 53.5 (SD = 5.87) vs. 50.83 (SD = 6.24) for the others, and the mean MCS score for the primary prophylaxis group was 47.2 (SD = 8.90) vs 52.4 (SD = 7.80). Data for the combined sample are shown in Fig. 1, and may suggest that primary prophylaxis is associated with better physical scores but worse mental scores. It is worth noting that the group on primary prophylaxis were younger by approximately 5 years, were less likely to have a target joint, had lower (better) HJHS scores and all had severe hemophilia. We were not able to examine the relationship between those on primary prophylaxis with the disease-specific HRQoL scores because of small sample sizes.

SF-36 component summary scores were also examined according to weekly frequency of prophylactic regimen at the time of recruitment. In the combined cohort, the mean PCS score for the 8 subjects on prophylaxis one to two times per week was 53.6 (SD = 4.78). The 21 subjects on prophylaxis 3 times per week or alternate days had a mean PCS score of 52.4 (SD = 6.73). The 17 subjects receiving daily prophylaxis had a mean PCS score of 49.1 (SD = 5.66). Mean MCS scores for these same groups were 48.3 (SD = 9.9), 52.6 (SD = 8.0), and 51.0 (SD = 7.83) respectively.

### Impact of age

It is known that in the general population HRQoL diminishes as a function of age [5]. We found a reduction of 0.31 points per year of age (*p* = 0.14) in the PCS scores, and a decline by 0.33 per year (*p* = 0.25) in the MCS, but neither reached statistical significance. The reduction of scores as a function of age was also observed with the disease-specific measures: CHO-KLAT scores decreased by 0.73 points per year of age among the 13 youth (*p* = 0.78); and Haemo-QoL-A scores decreased 1.01 points per year of age (*p* = 0.09) among the 33 young adults.

## Discussion

This paper reports the baseline data of a study with a primary objective to describe the patterns of QoL over a 3-year period in Canadian youth and young adults who have moderate or severe hemophilia A and who are using Helixate FS®.

Forty-six subjects were recruited from 6 centres in 3 Canadian provinces but were predominantly from Quebec because Helixate FS® is preferentially used in that province for reasons of public tender contract. Although limited to one brand of recombinant factor concentrate, there is no reason to suspect that the product brand would have a significant impact on HRQoL. This cohort comprised subjects 12.9 to 28.7 years of age who were free from HIV or other significant comorbid conditions.

We had a relatively small group of youth, most of whom had been on prophylaxis from an early age, and a larger group of young adults most of whom had not been introduced to prophylaxis at an early age as is typical in Canada for these age groups. While very frequently used in Canada, prophylaxis is not universal in this age group and our findings may not be applicable to patients who are “on-demand”. There are no other recruitment criteria that would differentiate this cohort, and it is therefore a small but representative sample of Canadians with moderate or severe HA. The subjects were unremarkable in terms of their BMI scores. In our sample (mean age of 21.4 years), 52 % had significant joint disease (HJHS > 10) which compares to the slightly older Dutch cohort (mean age of 24.8 years) described by Fischer et al. [14], in which 46 % had significant joint disease.

### Generic HRQoL scores

The main focus of this study was on the HRQoL of youth and young adults with hemophilia which was captured using both a generic (SF-36) and two disease-specific questionnaires; one for youth (CHO-KLAT) and one for adults (Haemo-QoL-A). We began with an examination of the SF36 component scores. When we compared the results of our study subjects, between the ages of 16 to 24.9 years, to published normative data for healthy Canadian males in the same age range, we found that our mean scores were similar to the PCS and MCS norms. However, we found several notable differences when we examined the 8 domains within the SF-36 (see Table 2). Our cohort reported lower scores compared to Canadian normative data in the following domains: Physical Functioning (mean difference = 5.6), Role Physical (5.4), and Bodily Pain (9.9). The differences for Physical Functioning and Bodily Pain were clinically meaningful (exceeded the MIDs) and statistically significant. These results are consistent with what one would expect based on clinical experience. We also identified areas of strengths in our cohort, with higher scores than the norms in the following domains: Role Emotional (mean difference = 4.0) and Mental Health (2.9). These differences were not statistically significant.

Similar comparative analysis was conducted in Sweden, by Lindvall et al. [19], who reported on a cross-sectional study of severe HA subjects aged 15–34 years from a single treatment center. They found that SF-36 PCS and MCS scores were not statistically different than the national norms, which is consistent with our findings. However, they found significantly impaired HRQoL based on some of the SF-36 domains (PF and PR), and for PCS scores only in their older subjects (35 to 64 years). More intensive prophylaxis in Sweden than in Canada may explain the delay in decline of HRQoL in that country.

We found significant differences in PCS but not MCS scores related to joint status (HJHS) in our Canadian cohort. However, PCS and MCS were not worse in those reporting an active target joint in the preceding year, suggesting that functional joint status more than bleed frequency impacts HRQoL.

To explore the relationship of HRQoL to treatment history we first compared the PCS and MCS scores in those who had been on lifelong primary prophylaxis versus the others. We found better PCS scores but worse MCS scores in the primary prophylaxis group. These results should be interpreted with caution given the small number of subjects involved and the difference in age between the two categories of subjects. They are intriguing given that we identified Role Emotional and Mental Health domains as areas of strength in our hemophilia cohort compared to Canadian norms. The exploration of HRQoL in relation to prophylaxis frequency found an inverse relationship, in that those receiving the most frequent treatment (daily) had the lowest PCS scores. However, there was no clear relationship between frequency of treatment and MCS scores. These mental health findings warrant investigation in future larger studies.

When we explored age-related differences in the cross-sectional cohort, we found that PCS and MCS scores declined as a function of age in a similar fashion. However, clinical experience suggests that the PCS scores should decline to a greater extent than MCS, given the contributions of both the natural aging process and cumulative joint damage associated with hemophilia. This requires further examination in longitudinal studies.

### Disease specific HRQoL scores

The analysis of the disease-specific results from this study is limited due to the small sample sizes, particularly for youth. The mean CHO-KLAT score in this cohort (79.2) was somewhat higher than reported in previous studies such as: the Canadian validation study 74.6 [9]; Quebec validation study 71.9 [8]; and recent Toronto study 75.4 [20]. However, the scores are very close to the mean of 78.0 for a subset of 19 patients from the Toronto study who were 14 years of age or older [20].

Although the mean Haemo-QoL-A total score found in our young adults (85.8) appears high compared to the Rentz et al. results [10], our adult cohort was much younger, had less viral co-morbidity and did not include a significant proportion of subjects treated “on demand” (see Table 3). Similarly, Ingerslev found much higher Haemo-QoL-A total scores for Danish patients on prophylaxis (median score = 86.5) than in Russian patients that had been mostly treated on-demand (median score = 71.0). Manco-Johnson et al. [18] studied prospectively a cohort of severe HA subjects also age 14-29 years who had mostly been on prophylaxis. She reported a mean Haemo-QoL-A total score of 85.6 (SD = 10.7) for a group of 22 subjects (with a median age of 17 years) who had been on uninterrupted prophylaxis at the time of study entry. The results from the latter study are very similar to ours (Table 3).

Our data revealed modest correlations between disease specific scores and the generic SF36. The CHO-KLAT demonstrated a better relationship with the MCS than PCS and the Haemo-QoL-A demonstrated a better relationship with the PCS than MCS indicating again that they are not assessing the same aspects of quality of life.

We have found that global scores are less sensitive than subdomains in understanding HRQoL in severe hemophilia as a decline in physical scales may be offset by mental scores higher than normative data in this condition. The areas of bodily pain and physical functioning deserve particular attention when assessing differences in cohorts or treatment approach. The lack of statistical correlation between joint status and disease specific HRQoL scores (CHO-KLAT and Haemo-QoL-A) may reflect differences in the nature of the items included in these tools which focus more on emotional health.

We were not surprised to see the subtle decline in HRQoL as a function of age; this should be kept in mind when comparing SF-36 and other instruments’ scores between studies involving cohorts of different age groups. The small sample size did not allow us to perform more sophisticated analyses, therefore the relationship between age, HJHS and HRQoL could not be fully assessed.

## Conclusions

Despite having mostly severe hemophilia, the youth in this cohort had minimal joint disease and good HRQoL. The young adults demonstrated more joint disease and slightly worse HRQoL – but were almost comparable to healthy populations of the same age. These findings confirm those of other investigators studying cohorts in developed countries and should now be expected in patients with access to adequate levels of prophylaxis.

We also identified that primary prophylaxis was associated with better PCS scores, which is consistent with their younger age, lower prevalence of target joints and better HJHS scores. However, this group also had lower MCS scores which were atypical for their age group. This requires further examination and potentially warrants more emotional health related questions be asked of youth with a history of primary prophylaxis in clinical practice.

Comparatively little is known about factors that might influence longitudinal patterns of HRQoL in patients on a stable treatment regimen. The current results form the foundation for a longitudinal study to examine the impact of biological factors and life events on the HRQoL of youth and young adults with haemophilia followed prospectively for 3 years.

## Ethics approval and consent to participate

Written informed consent was provided by all subjects age 16 and above prior to enrollment. In subjects under age 16 assent was documented in addition to written consent of legal guardian. The study was approved by the Ethics Review Boards (ERB) of all participating institutions (see list of ERB in Additional file 1) and complied with the guidelines of Good Clinical Practice.

## Consent for publication

Not applicable.

## Availability of data and materials

Data will not be made available in order to protect the participants’ identities.

## Additional file

Additional file 1: Full name of all the Research Ethics Boards which approved the study. (DOCX 14 kb)

## Abbreviations

BP: Bodily Pain
CHO-KLAT: Canadian Haemophilia Outcomes – Kids Life Assessment Tool
ERB: Ethics Review Board
FVIII: Factor VIII
GH: General Health
HA: Hemophilia A
HCV: Hepatitis C virus
HIV: Human immunodeficiency virus
HJHS: Hemophilia Joint Health Score (HJHS) version 2.0
HRQoL: Health-related quality of life
MCS: Mental Component Summary
MH: Mental Health
PCS: Physical Component Summary
PF: Physical Functioning
RF: Role Emotion
RP: Role Physical
SD: Standard Deviation
SF: Social Functioning
SF-36: Short Form 36
VT: Vitality
