# Supplementary material for: Generic and disease-specific quality of life among youth and young men with Hemophilia in Canada
Source: BMC Hematol. 2016 May 5;16:13. doi: 10.1186/s12878-016-0052-x (PMC4858890; doi:10.1186/s12878-016-0052-x)
Supplement: Additional file 1: — Full name of all the Research Ethics Boards which approved the study. (DOCX 14 kb) [file 12878_2016_52_MOESM1_ESM.docx]

**Generic and Disease-Specific Quality of Life**

**Among Youth and Young Men with Hemophilia in Canada.**

**Supplementary File:**

**Full name of all the Research Ethics Boards which approved the study**

Comité d’éthique de la recherche

Centre Hospitalier Universitaire Sainte-Justine

3175, Côte-Ste-Catherine Road

Montréal, QC, Canada, H3T 1C5

Ethics Review Board

Laurentian University

935 Ramsey Lake Road

Sudbury, Ontario, Canada, P3E 2C6

Research Ethics Board of the Hospital for Sick Children

The Hospital for Sick Children

555 University Avenue

Toronto, ON, Canada, M5G 1X8

Health Research Ethics Board of the University of Alberta

University of Alberta

4-71 Medical Science Building

Edmonton, Alberta, Canada, T6G 2H7

Children’s Hospital of Eastern Ontario Research Ethics Board

Children’s Hospital of Eastern Ontario

401 Smyth Road

Ottawa, ON, Canada, K1H 8L1

The Ottawa Hospital Research Ethics Board

The Ottawa Hospital

501 Smyth Road

Ottawa, ON, Canada, K1H 8L6

Research Ethics Board

The Research Institute of the McGill University Health Center

2155 Guy Street,

Montreal, QC, Canada, H3H 2R9

Comité d’éthique de la recherche

Centre hospitalier affilié universitaire de Québec (CHA)

1401, 18th Street

Québec City, QC, Canada, G1J 1Z4
